# Association of General Anesthesia and Neuraxial Anesthesia in Caesarean Section with Maternal Postpartum Depression: A Retrospective Nationwide Population-Based Cohort Study

**DOI:** 10.3390/jpm12060970

**Published:** 2022-06-14

**Authors:** Kuo-Hsun Hung, Shao-Lun Tsao, Shun-Fa Yang, Bo-Yuan Wang, Jing-Yang Huang, Wen-Tyng Li, Liang-Tsai Yeh, Cheng-Hung Lin, Yin-Yang Chen, Chao-Bin Yeh

**Affiliations:** 1 Institute of Medicine, Chung Shan Medical University, Taichung 402, Taiwan; paulhung13@gmail.com (K.-H.H.); ysf@csmu.edu.tw (S.-F.Y.); chibochichiyasu@gmail.com (B.-Y.W.); wchinyang@gmail.com (J.-Y.H.); 68990@cch.org.tw (L.-T.Y.); 2 Department of Biomedical Engineering, Chung Yuan Christian University, Taoyuan 320, Taiwan; 117223@cch.org.tw (S.-L.T.); wtli@cycu.edu.tw (W.-T.L.); 3 Department of Anesthesiology, Changhua Christian Hospital, Changhua 500, Taiwan; 4 Department of Medical Research, Chung Shan Medical University Hospital, Taichung 402, Taiwan; 5 Department of Emergency Medicine, School of Medicine, Chung Shan Medical University, Taichung 402, Taiwan; 6 Department of Emergency Medicine, Chung Shan Medical University Hospital, Taichung 402, Taiwan; 7 Department of Post-Baccalaureate Medicine, College of Medicine, National Chung Hsing University, Taichung 402, Taiwan; 8 Department of Information Technology, Chung Shan Medical University Hospital, Taichung 402, Taiwan; cshe563@csh.org.tw; 9 Department of Medical Informatics, Chung Shan Medical University, Taichung 402, Taiwan; 10 Department of Surgery, Chung Shan Medical University Hospital, Taichung 402, Taiwan

**Keywords:** cesarean section, general anesthesia, neuraxial anesthesia, postpartum depression

## Abstract

Although cesarean section (CS) has become a common method of child delivery in recent decades, the choice between general anesthesia (GA) and neuraxial anesthesia (NA) for CS must be carefully considered. Depending on the type of anesthesia used in CS, a major outcome observed is the occurrence of postpartum depression (PPD). This study investigated the association between PPD risk and the anesthesia method used in CS by using data from three linked nationwide databases in Taiwan, namely, the National Health Insurance Research Database, the National Birth Reporting Database, and the National Death Index Database. After propensity score matching by baseline depressive disorders, maternal demographics, status at delivery, infant’s health, maternal diseases during pregnancy, and age of partner, we included women who had natural births (*n* = 15,706), cesarean sections with GA (*n* = 15,706), and cesarean sections with NA (*n* = 15,706). A conditional logistic regression was used to estimate the odds ratios and 95% confidence intervals (CIs) of PPDs, including depression, sleep disorder, and medication with hypnotics or antidepressants, under anesthesia during CS. The prevalence rates of combined PPDs were 26.66%, 43.87%, and 36.30% in natural births, CS with GA, and CS with NA, respectively. In particular, the proportions of postpartum use of hypnotic drugs or antidepressants were 21.70%, 39.77%, and 31.84%, which were significantly different. The aORs (95% CIs) were 2.15 (2.05–2.25) for the included depressive disorders, 1.10 (1.00–1.21) for depression, 1.03 (0.96–1.11) for sleep disorder, and 2.38 (2.27–2.50) for medication with hypnotics or antidepressants in CS with GA compared with natural births. Women who underwent CS with GA had a significantly higher risk of depressive disorders and a higher need for antidepressants for sleep problems than those who underwent CS with NA. The risks of PPD were significantly associated with the anesthesia method, especially GA. Our results can assist physicians in carefully considering the appropriate anesthesia method for CS delivery, particularly with regard to postpartum drug abuse and drug safety.

## 1. Introduction

Cesarean section (CS) is among the most common surgical procedures, with a global rate of 18.6% [1]. In Taiwan, the CS rate has exceeded 30% in the past decade [2]. The main purpose of CS is as a life-saving procedure that improves the survival chances of both the mother and baby. With advances in medical technology and changes in social perception, CS has gradually become a common delivery route. However, CS entails adverse effects such as increased risks of maternal complications, including infection, postpartum hemorrhage, ureter and bladder injury, uterine rupture, chronic pelvic pain, gastrointestinal dysfunction, and rehospitalization [3].

Two main types of anesthesia are employed in CS, namely, general anesthesia (GA) and neuraxial anesthesia (NA). In modern obstetric practice, NA is the preferred method of anesthesia in terms of risk and benefits for both the mother and fetus [4,5,6]. However, in certain emergent situations (e.g., fetal bradycardia, maternal hemorrhage, and uterine rupture), GA may be required for cesarean delivery because it is rapid and reliable, allowing for airway control and improved hemodynamic stability, compared with NA. Before performing a CS, anesthesiologists must identify any comorbidities and evaluate the possible outcomes of each anesthesia method. In emergencies, providing safe anesthesia under adequate monitoring is critical [7,8,9].

Postpartum depression (PPD) is the most common maternal health problem after delivery. The prevalence of PPD varies between countries, from nearly 0% to as high as 60%. PPD can have adverse effects on both the mother and child, including diminished maternal–infant contact and infant feeding and increased risks of self-harm, suicidal ideation, and infanticide. Maternal suicide is the leading cause of death during the postpartum period in the United Kingdom; furthermore, in the United States, the rate of depressive disorders recorded during hospitalization for delivery has increased [10,11]. With symptoms typically beginning 1 month after delivery, PPD is a depressive episode that occurs within 1 year after delivery. Several epidemiological studies conducted during the last three decades have examined the association between CS and PPD. A meta-analysis conducted in China that included 532,630 participants showed that the pooled OR of the association between CS and the risk of PPD was 1.26 (95% CI 1.16–1.36). Furthermore, emergency CS was associated with a higher risk of PPD than elective CS, with pooled ORs of PPD of 1.47 (95% CI 1.33–1.62) and 1.15 (95% CI 0.92–1.43), respectively [3]. Another meta-analysis that included 32 articles also reported that compared to vaginal delivery CS is associated with an increased risk of PPD (adjusted OR, 1.15, 95% CI 1.00–1.34), and emergency CS is associated with a higher risk of PPD [12]. However, the results have not been convincing. Many of these studies did not consider major risk factors associated with PPD, including sociodemographic factors, pregnancy-related characteristics, newborn-related characteristics, and maternal psychological characteristics [13,14,15], when examining the effects of the method of anesthesia used in CS on the risk of PPD. Therefore, this study investigated the association between PPD occurrence within 1 year after labor and the method of anesthesia used in CS by analyzing data from the Longitudinal Health Insurance Database in Taiwan.

## 2. Materials and Methods

### 2.1. Data Source

We conducted a retrospective nested case-control study by analyzing data from between 2008 and 2017 from three linked nationwide databases, namely, the National Health Insurance Research Database (NHIRD), the National Birth Reporting Database, and the National Death Index Database, in Taiwan. The datasets are maintained for academic research by Taiwan’s Health and Welfare Data Science Center [16]. Disease diagnosis was obtained from medical claims records. Information on medical claims of inpatient and outpatient visits to clinics or hospitals (diagnostic codes, date of visit, prescription, and medical orders) and data on patients’ date of birth and parental monthly income were obtained from the NHIRD. Information on the characteristics of mothers, infants, and mothers’ deliveries was obtained from the National Birth Reporting Database. The extracted data included the method of delivery, infant sex, birth weight, and gestational age. Information on the survival status of infants was obtained from the National Death Index Database.

All residents in Taiwan have a unique personal identification number, which permits the linking of information across nationwide databases. The relationship between parents and children was confirmed through data linkage with the birth registration system in Taiwan, the details of which can be found in previous reports [17]. The Institutional Review Board of Chung Shan Medical University Hospital approved the study (CS1-20004). All data were encrypted and remained anonymous during data analysis.

### 2.2. Study Population

**Figure 1:**
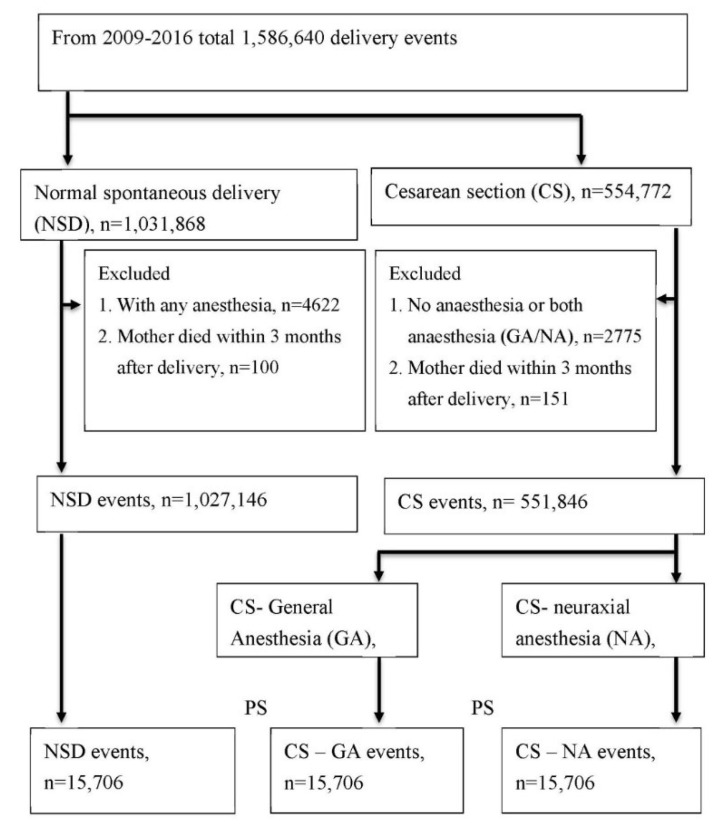
Flow charts for sample selection.

Initially, 1,586,640 labor events, including 1,031,868 natural births and 554,772 (35%) CSs between 1 January 2008 and 31 December 2016 were identified. We excluded the cases of anesthesia during natural birth (*n* = 4622), both GA and NA performed during admission for CS (*n* = 2775), and death within 3 months after labor (*n* = 251). Figure 1 illustrates the flowchart of patient selection. Next, we classified labor events into three groups, namely, natural birth (*n* = 1,027,146), CS with GA (*n* = 16,234), and CS with NA (*n* = 535,612). The type of anesthesia performed was obtained from the NHIRD. GA was identified by the order codes 96,017, 96,018, 96,019, 96,020, 96,021, and 96,022, and NA was defined by the order codes 96,007, 96,008, 96,005, and 96,006.

### 2.3. Propensity Score Matching and Study Covariates

To balance the baseline characteristics (i.e., within 1 year before pregnancy or during pregnancy) and the health status of offspring within 3 months after birth, we estimated the average treatment effect of CS with GA and then selected the propensity-score-matched natural birth and CS with NA events by using a greedy nearest-neighbor matching algorithm and nonreplacement paired within 0.01 caliper widths.

In propensity score matching, we considered the following covariates: baseline depression; sleep disorders and hypnotic medication, sedatives, or antidepressants; maternal sociodemographic characteristics (i.e., nationality, mother age, urbanization, and insurance amount); status at delivery (i.e., year of delivery, stillbirth, sex of infant, gestational age, infant’s birth weight, and Apgar score at 1 min); newborn-related outcomes (i.e., death within 1 year after birth, tube feeding, use of ventilator, intensive care unit admission, diagnosis of birth defect, cardiovascular disease, central nervous system disease, respiratory disease, or gastrointestinal disease); maternal psychosocial characteristics during pregnancy; and age of partner.

### 2.4. Definition of Postpartum Depressive Disorders

In this study, the date of birth and gestational age were used to define the perinatal period (three periods, namely, 1 year before pregnancy, during pregnancy, and 1 year postpartum). Moreover, we identified depressive disorders that constituted the diagnoses of depression (*International Classification of Diseases, Ninth Revision, Clinical Modification* (*ICD-9-CM*) codes 296, 300, 308, 309, 311, and 648.4 and *International Classification of Diseases, Tenth Revision, Clinical Modification* (*ICD-10-CM*) codes F30–F34, F40–F45, O99.3, O99.6, and R45 [18], sleep disorder (*ICD-9-CM* codes 327, 347, 307.4, 770.8, 780.5, and V69.4 and *ICD-10-CM* codes F51, G47, and Z72.8) [19], and medication with hypnotics (Anatomical Therapeutic Chemical Classification (ATC) code N05C) or antidepressants (ATC code N06A) during the perinatal period.

### 2.5. Statistical Analysis

An analysis using a large sample size is likely to reveal a statistically significant difference of *p* < 0.05, even if the effect size is negligible or small [20]. We used an absolute standardized difference (ASD) to compare the statistical values of baseline covariates between groups in this large-sample observational study. The characteristics were balanced when the ASD was <0.1. A conditional logistic regression was used to estimate odds ratios and 95% confidence intervals (CIs) of PPD disorders, including depression, sleep disorder, and medication with hypnotics or antidepressants, under anesthesia during CS. SAS version 9.4 (SAS Institute, Cary, NC, USA) was used for the statistical analysis. The significance level was set at 0.05, and a two-tailed test was used.

## 3. Results

### 3.1. Group Characteristics after Propensity Score Matching

After propensity score matching, we identified 15,706 women who had natural births, CS with GA, and CS with NA. The baseline maternal demographics, status at delivery, infant death within 1 year after birth, infant health, maternal diseases during pregnancy, and age of partner were balanced with a standardized difference of <0.1 (Table 1).

### 3.2. Depressive Disorders during the Perinatal Period

Table 2 lists depressive disorders during the perinatal period after propensity score matching. Within 1 year before pregnancy, the prevalence rates of depressive disorders were 31.19%, 31.03%, and 30.93% for natural birth, GA, and NA, respectively. During pregnancy (antenatal), the prevalence rates of depressive disorders were 16.96%, 17.55%, and 17.48% for natural birth, GA, and NA, respectively. After labor, the prevalence rates of depressive disorders were 26.66%, 43.87%, and 36.30% for natural birth, GA, and NA, respectively. The rates of the postpartum use of hypnotics or antidepressants were 21.70%, 39.77%, and 31.84%, respectively, which were significantly different.

**Figure 2:**
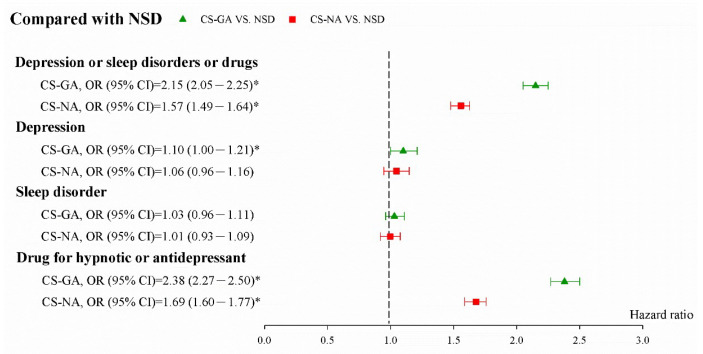
aOR (95% CI) of studied events in CS with GA compared with natural birth after propensity score matching. NSD: Normal Spontaneous Delivery; CS-GA: Cesarean Section—General Anesthesia; CS-NA: Cesarean Section—Neuraxial Anesthesia. * Indicates *p* < 0.05.

**Figure 3:**
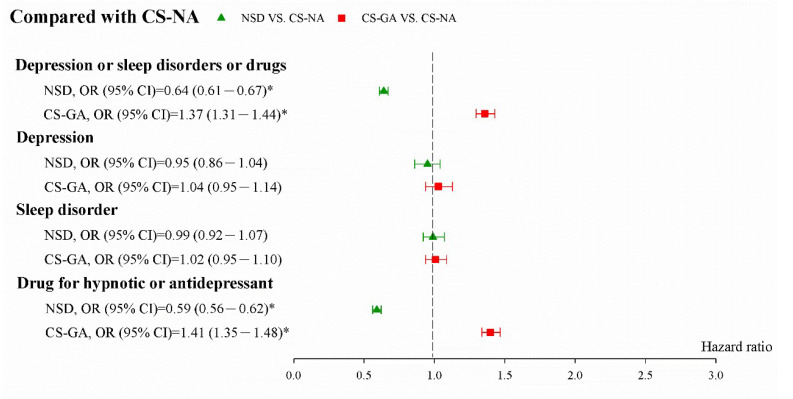
aOR (95% CI) of studied events of CS with GA compared with CS with NA after propensity score matching. NSD: Normal Spontaneous Delivery; CS-GA: Cesarean Section—General Anesthesia; CS-NA: Cesarean Section—Neuraxial Anesthesia. * Indicates *p* < 0.05.

Figure 2 displays the adjusted ORs (aORs) and 95% CIs of combined PPDs. The aOR (95% CI) was 2.15 (2.05–2.25) for all depressive disorders, 1.10 (1.00–1.21) for depression, 1.03 (0.96–1.11) for sleep disorder, and 2.38 (2.27–2.50) for the postpartum use of hypnotic or antidepressant drugs in CS with GA compared with natural birth. Figure 3 shows the aOR (95% CI) was 1.37 (1.31–1.44) for combined depressive disorders, 1.04 (0.95–1.14) for depression, 1.02 (0.95–1.10) for sleep disorders, and 1.41 (1.35–1.48) for the postpartum use of hypnotic drugs or antidepressant drugs in CS with GA compared with CS with NA.

We examined mothers who had no depressive disorder at baseline. Compared with natural birth, CS with GA had the highest risk of PPDs (aOR = 2.49, 95% CI = 2.33–2.65) and postpartum use of hypnotic drugs or antidepressant drugs (aOR = 2.84, 95% CI = 2.65–3.04), as shown in Table 3. Supplementary Table S1 presents the results of the analysis, stratified by subgroups (including mother’s age, father’s age, gestational age, infant’s birth weight, urbanization, and insurance amount). We obtained similar results for all stratified subgroup analyses for the risk of postpartum depression in the population matched by propensity score.

## 4. Discussion

Few studies have determined the association between the occurrence of PPD and GA for CS compared with that of NA; however, the relationship between the anesthesia method and PPD risk remains unclear. In our study, which analyzed data from the Taiwan Longitudinal Health Insurance Database, we observed that the use of GA in CS delivery was associated with significantly higher risks of PPD and sleep disorders and entailed the medical prescription of antidepressants for sleep problems in the postpartum period compared with the use of NA (aOR, 1.37; 95% CI, 1.31–1.44). Our study revealed that GA was associated with a 139% increase in the risk of PPD (OR, 2.393; 95% CI, 2.314–2.474), and NA involved a 74% increase in the risk of PPD (OR, 1.74; 95% CI, 1.73–1.76) compared with normal spontaneous delivery. Jean et al. reported that GA was associated with a 54% increase in the risk of PPD, including cases requiring hospitalization, suicidal ideation, and self-injury [10], a finding consistent with that of our study.

The association between GA, surgery, and postoperative depression is not well established, and studies have largely focused on the characteristics of obstetric patients. Generally, risk factors for PPD involve patients’ sociodemographic, individual, and delivery characteristics [21], including poor marital relationship, prenatal depression, child illness, low socioeconomic status, low educational level, unwanted pregnancy, obesity, previous history of PPD, physical symptoms [22], and perioperative events [23,24]. CS may similarly adversely affect psychological outcomes because surgical trauma can induce a stress response in mothers, thereby increasing the risk of PPD [25]. Our study revealed that, compared with vaginal birth, CS was significantly associated with PPD. These findings are consistent with those of two meta-analyses, which have indicated that, compared with vaginal birth, CS is associated with an increased risk of PPD [2,12]. When GA is performed in CS, opioids and benzodiazepines are typically administered after the baby is delivered to prevent the transfer of these drugs to the newborn through the placenta. Few studies have discussed the continued use of antidepressants and hypnotics after delivery. This study revealed that the postpartum use of antidepressants and hypnotics was the highest in the GA population, drawing attention to the adverse effects of improper drug use on mothers and infants [26,27,28].

The association between anesthesia and PPD was widely studied in previous studies. The Japan Environment and Children’s Study (JECS), a prospective cohort study that enrolled registered fetal records (*n* = 104,065) in 15 regions nationwide in Japan, showed that vaginal delivery with anesthesia was associated with a higher risk of PPD (aOR: 1.233, 95% CI 1.079–1.409) [29]. Edipoglu et al. conducted another observational study in Turkey of 92 patients and showed that epidural analgesia was strongly correlated with lower depression scores (OR = 0.29, *p* = 0.0001) [30]. Furthermore, Lim et al. organized a single institutional retrospective observational study on epidural analgesia and the risk of PPD and showed that epidural analgesia was associated with a reduction in the severity of depression symptoms [31]. Our study provided a more precise aspect of the association between different anesthesias and PPD in CS patients and showed a similar result of a lower risk of PPD in patients with NA compared to GA.

The CS rate varies widely throughout the world, with 21.1% caesarean in the world, 31.6 in North America, 25.7 in Europe, and 23.1 in Asia [32]. In Taiwan, according to the report of the Ministry of Health and Welfare of Health Promotion Administration, CS rates were above 30% during the past decade. Owing to the coding system of the NHIRD, the CS cases enrolled in our study included both elective and emergency CS. However, empirically, GA was employed for emergency CS instead of NA for women with severe maternal morbidity (SMM), such as a major hemorrhage or abnormal placentation, which could cause confounding effects, because GA has been demonstrated to be associated with PPD and post-traumatic stress disorder [33,34]. For scheduled CS, Abe et al. performed a nationwide population-based study to determine the association between the anesthesia method and SMM in a Japanese population and reported that GA was associated with increased SMM compared with NA (OR, 2.68; 95% CI, 1.97–3.64) [35]. Another population-based cross-sectional study including 575, 524 women demonstrated that NA for vaginal delivery was associated with a 14% decrease in the risk of SMM [36] and hence a lower risk of PPD.

Our study provided additional information, including that of neonatal outcomes, outpatient or emergency treatment data for PPD, and records of antidepressant prescriptions. Such information was not reported by previous studies, which might have resulted in underestimating the real incidence of PPD. Almost all births in Taiwan are attended by obstetricians and are registered in the National Health Insurance Database, and all clinical visits with prescription drugs for PPD and sleep disorders are recorded in the database. Our study may offer a more comprehensive and accurate analysis of the occurrence of PPD than previous studies. In this study, newborns from mothers who had GA during CS had a higher percentage of poor health, the requirement of more medical support, and death within 1 month after birth than newborns from mothers who had NA. The health condition of such children may cause stress to their mothers, thereby leading to mood disorders in the postpartum period [37]. With the gradual increase in CS in past decades, the choice between GA and NA in CS not only represents the anesthesia method used for CS but also its subsequent effects on the mother and child. Our findings provide information on the relationship between GA in CS and PPD, which can help women make better anesthesia choices for CS. The causes of PPD are multiple and complicated, and some risk factors for PPD cannot be avoided during the perinatal period. Under the permissible medical conditions, mothers can make the most appropriate choice of anesthesia for the caesarean section based on research.

This study has some limitations. First, the NHIRD does not provide information on lifestyle or personal behavioral factors, including smoking, alcohol consumption, and body mass index, which might affect the risk of maternal complications and PPD. Second, the coding system of the NHIRD could not reveal whether CS was emergent or elective. Patients requiring emergency CS might have severe maternal complications, and this could lead to an unpleasant birth process, further causing depressive disorders. Where possible, we adjusted and matched baseline maternal sociodemographic characteristics, delivery status, neonatal characteristics, maternal psychosocial characteristics during pregnancy, and the ages of partners between the different anesthesia groups to overcome this bias. Third, no specific code for PPD exists in the NHIRD, and we explored every possible means to obtain relevant information that can indicate the occurrence of PPD in clinic settings. Nevertheless, the true incidence of PPD might still be underestimated.

## 5. Conclusions

In conclusion, our findings can help psychiatrists and obstetricians to identify high-risk populations for PPD, thus enabling them to arrange consultation and intervention with them as early as possible. Puerpera who must choose GA as the anesthesia method for CS should be routinely informed regarding PPD to help recognize mood disturbances during the postpartum period and seek help before it is too late.

## Figures and Tables

**Table 1:** Characteristics of participants after propensity score matching.

Variables	NSD	CS-GA	CS-NA
Number (*n*)	15,706	15,706	15,706
Mother death within 3 months to 1 year after delivery	5 (0.03%)	12 (0.08%)	3 (0.02%)
Childbirth year			
2009	2310 (14.71%)	2171 (13.82%)	2318 (14.76%)
2010	1974 (12.57%)	1891 (12.04%)	2035 (12.96%)
2011	1859 (11.84%)	1849 (11.77%)	1884 (12.00%)
2012	2153 (13.71%)	2113 (13.45%)	2136 (13.60%)
2013	1752 (11.15%)	1781 (11.34%)	1746 (11.12%)
2014	1849 (11.77%)	1889 (12.03%)	1878 (11.96%)
2015	1914 (12.19%)	1937 (12.33%)	1828 (11.64%)
2016	1895 (12.07%)	2075 (13.21%)	1881 (11.98%)
Delivery within 1 year before this time	140 (0.89%)	160 (1.02%)	143 (0.91%)
Anesthesia within 1 year before this time			
GA	239 (1.52%)	246 (1.57%)	238 (1.52%)
NA	234 (1.49%)	227 (1.45%)	201 (1.28%)
Obstetric History			
Primigravida	14,958 (95.24%)	14,851 (94.56%)	14,755 (93.94%)
Multigravida	748 (4.76%)	855 (5.44%)	951 (6.06%)
Country of mother			
Domestic	15,163 (96.54%)	15,146 (96.43%)	15,128 (96.32%)
Foreign	543 (3.46%)	560 (3.57%)	578 (3.68%)
Mother age in delivery (y/o)			
<25	940 (5.98%)	1012 (6.44%)	999 (6.36%)
25–30	2898 (18.45%)	2961 (18.85%)	2944 (18.74%)
30–35	6260 (39.86%)	6291 (40.05%)	6267 (39.90%)
35–40	4453 (28.35%)	4361 (27.77%)	4420 (28.14%)
>40	1155 (7.35%)	1081 (6.88%)	1076 (6.85%)
Urbanization			
Urban	9659 (61.50%)	9526 (60.65%)	9502 (60.50%)
Suburban	4838 (30.80%)	4960 (31.58%)	4977 (31.69%)
Rural	1209 (7.70%)	1220 (7.77%)	1227 (7.81%)
Insurance amount			
<20,000	5848 (37.23%)	5873 (37.39%)	5775 (36.77%)
20,000–50,000	8880 (56.54%)	8909 (56.72%)	9014 (57.39%)
>50,000	978 (6.23%)	924 (5.88%)	917 (5.84%)
Stillbirth or miscarriage	683 (4.35%)	344 (2.19%)	277 (1.76%)
Male baby	8601 (54.76%)	8560 (54.50%)	8559 (54.50%)
Gestational weeks			
Preterm labor (<28 weeks)	827 (5.27%)	704 (4.48%)	537 (3.42%)
Preterm labor (28–32 weeks)	902 (5.74%)	1034 (6.58%)	991 (6.31%)
Preterm labor (32–37 weeks)	2439 (15.53%)	3613 (23.00%)	3335 (21.23%)
Full term labor (37–42 weeks)	11,525 (73.38%)	10,341 (65.84%)	10,830 (68.95%)
Full term labor (>42 weeks)	13 (0.08%)	14 (0.09%)	13 (0.08%)
Birth weight (gm)			
<1000	709 (4.51%)	794 (5.06%)	717 (4.57%)
1000–1500	797 (5.07%)	869 (5.53%)	877 (5.58%)
1500–2500	3170 (20.18%)	3172 (20.20%)	3355 (21.36%)
2500–3500	9054 (57.65%)	8971 (57.12%)	8883 (56.56%)
>3500	1976 (12.58%)	1900 (12.10%)	1874 (11.93%)
Apgar score at 1 min <5	2852 (18.16%)	3023 (19.25%)	2861 (18.22%)
Missing of offspring’s ID	1572 (10.01%)	1302 (8.29%)	1299 (8.27%)
Offspring death within 1 year after delivery	213 (1.36%)	217 (1.38%)	210 (1.34%)
Baby within 3 months after birth			
NG tube feeding treatment	4157 (26.47%)	4133 (26.31%)	4191 (26.68%)
Ventilation-assisted treatment	6426 (40.91%)	6347 (40.41%)	6556 (41.74%)
Intensive care unit	4309 (27.44%)	4300 (27.38%)	4343 (27.65%)
Infant’s disease within 3 months after birth			
Congenital defect	1677 (10.68%)	1637 (10.42%)	1652 (10.52%)
Neonatal heart disease-related	992 (6.32%)	960 (6.11%)	943 (6.00%)
Neonatal neurology disease-related	233 (1.48%)	251 (1.60%)	241 (1.53%)
Neonatal respiratory disease-related	1365 (8.69%)	1418 (9.03%)	1376 (8.76%)
Neonatal gastrointestinal symptoms	715 (4.55%)	779 (4.96%)	741 (4.72%)
Mother disease during pregnancy			
Liver disease	408 (2.60%)	387 (2.46%)	374 (2.38%)
Kidney disease	120 (0.76%)	141 (0.90%)	129 (0.82%)
Rheumatic disease	165 (1.05%)	159 (1.01%)	154 (0.98%)
Chronic pulmonary diseases	549 (3.50%)	565 (3.60%)	570 (3.63%)
Urinary tract infection	2643 (16.83%)	2656 (16.91%)	2591 (16.50%)
Pneumonia	385 (2.45%)	389 (2.48%)	375 (2.39%)
Ectopic pregnancy and hydatidiform mole	1069 (6.81%)	1091 (6.95%)	1101 (7.01%)
Complications during labor	5737 (36.53%)	5263 (33.51%)	5132 (32.68%)
Anemia	4479 (28.52%)	4241 (27.00%)	4384 (27.91%)
Epilepsy	139 (0.89%)	133 (0.85%)	125 (0.80%)
Asthma	309 (1.97%)	322 (2.05%)	331 (2.11%)
Eclampsia or preeclampsia	2281 (14.52%)	2446 (15.57%)	2451 (15.61%)
Hypertension	1204 (7.67%)	1318 (8.39%)	1340 (8.53%)
GDM	1322 (8.42%)	1349 (8.59%)	1324 (8.43%)
Diabetes mellitus	841 (5.35%)	827 (5.27%)	853 (5.43%)
Thyroid dysfunction	621 (3.95%)	633 (4.03%)	623 (3.97%)
Father age at childbirth (y/o)			
Missing	2348 (14.95%)	2262 (14.40%)	2218 (14.12%)
<25	337 (2.15%)	350 (2.23%)	357 (2.27%)
25–30	1559 (9.93%)	1657 (10.55%)	1616 (10.29%)
30–35	4617 (29.40%)	4631 (29.49%)	4664 (29.70%)
35–40	4355 (27.73%)	4331 (27.58%)	4328 (27.56%)
>40	2490 (15.85%)	2475 (15.76%)	2523 (16.06%)

NSD: Normal Spontaneous Delivery; CS-GA: Cesarean Section—General Anesthesia; CS-NA: Cesarean Section—Neuraxial Anesthesia.

**Table 2:** Depressive disorders during perinatal period.

Variables	NSD	CS-GA	CS-NA
Number (*n*)	15,706	15,706	15,706
Within 1 year before pregnancy			
Depression, sleep disorder, or hypnotic and antidepressant drugs	4898 (31.19%)	4873 (31.03%)	4858 (30.93%)
Depression	1359 (8.65%)	1345 (8.56%)	1319 (8.40%)
Sleep disorder	1880 (11.97%)	1837 (11.70%)	1798 (11.45%)
Hypnotic and antidepressant drugs	3985 (25.37%)	3904 (24.86%)	3986 (25.38%)
During pregnancy			
Depression, sleep disorder, or hypnotic and antidepressant drugs	2664 (16.96%)	2757 (17.55%)	2745 (17.48%)
Depression	770 (4.90%)	783 (4.99%)	786 (5.00%)
Sleep disorder	913 (5.81%)	947 (6.03%)	954 (6.07%)
Hypnotic and antidepressant drugs	1907 (12.14%)	1945 (12.38%)	1948 (12.40%)
1 year postpartum			
Depression, sleep disorder, or hypnotic and antidepressant drugs	4187 (26.66%)	6890 (43.87%)	5701 (36.30%)
Depression	928 (5.91%)	1014 (6.46%)	977 (6.22%)
Sleep disorder	1444 (9.19%)	1486 (9.46%)	1455 (9.26%)
Hypnotic and antidepressant drugs	3408 (21.70%)	6247 (39.77%)	5001 (31.84%)

**Table 3:** aOR (95% CI) of studied events in mothers without postpartum depression, sleep disorder, hypnotics, or antidepressant drugs at baseline.

Variables	NSD	CS-GA	CS-NA
Postpartum depression, sleep disorder, hypnotics, or antidepressant drugs	Reference	2.49 (2.33–2.65) *	1.70 (1.59–1.82) *
Postpartum depression	Reference	1.17 (0.99–1.39)	1.18 (1.00–1.39)
Postpartum sleep disorder	Reference	1.03 (0.91–1.16)	1.04 (0.92–1.18)
Postpartum use of hypnotics or antidepressants drugs	Reference	2.84 (2.65–3.04) *	1.85 (1.72–1.98) *

NSD: Normal Spontaneous Delivery; CS-GA: Cesarean Section—General Anesthesia; CS-NA: Cesarean Section—Neuraxial Anesthesia. * Indicates *p* < 0.05.

## Data Availability

Restrictions apply to the availability of these data. Data were obtained from National Health Insurance database and are available from the authors with the permission of National Health Insurance Administration of Taiwan.

## Supplementary Materials

The following supporting information can be downloaded at: https://www.mdpi.com/article/10.3390/jpm12060970/s1, Table S1: Subgroup analysis for the risk of postpartum depression in propensity-score-matched population

## References

[1] Yokoyama M., Tanaka K., Sugiyama T., Arakawa M., Miyake Y. (2021). Cesarean section is associated with increased risk of postpartum depressive symptoms in Japan: The kyushu okinawa maternal and child health study. J. Affect. Disord..

[2] Chen H.H., Lai J.C., Hwang S.J., Huang N., Chou Y.J., Chien L.Y. (2017). Understanding the relationship between cesarean birth and stress, anxiety, and depression after childbirth: A nationwide cohort study. Birth.

[3] Xu H., Ding Y., Ma Y., Xin X., Zhang D. (2017). Cesarean section and risk of postpartum depression: A meta-analysis. J. Psychosom. Res..

[4] Maheshwari D., Ismail S. (2015). Preoperative anxiety in patients selecting either general or regional anesthesia for elective cesarean section. J. Anaesthesiol. Clin. Pharmacol..

[5] Yurashevich M., Carvalho B., Butwick A.J., Ando K., Flood P.D. (2019). Determinants of women’s dissatisfaction with anaesthesia care in labour and delivery. Anaesthesia.

[6] Nikolajsen L., Sørensen H.C., Jensen T.S., Kehlet H. (2004). Chronic pain following caesarean section. Acta Anaesthesiol. Scand..

[7] Rollins M., Lucero J. (2012). Overview of anesthetic considerations for cesarean delivery. Br. Med. Bull..

[8] Guglielminotti J., Landau R., Li G. (2019). Adverse events and factors associated with potentially avoidable use of general anesthesia in cesarean deliveries. Anesthesiology.

[9] Yeh H.W., Yeh L.T., Chou Y.H., Yang S.F., Ho S.W., Yeh Y.T., Yeh Y.T., Wang Y.H., Chan C.H., Yeh C.B. (2019). Risk of cardiovascular disease due to general anesthesia and neuraxial anesthesia in lower-limb fracture patients: A retrospective population-based cohort study. Int. J. Environ. Res. Public Health.

[10] Guglielminotti J., Li G. (2020). Exposure to general anesthesia for cesarean delivery and odds of severe postpartum depression requiring hospitalization. Anesth. Analg..

[11] Haight S.C., Byatt N., Moore Simas T.A., Robbins C.L., Ko J.Y. (2019). Recorded diagnoses of depression during delivery hospitalizations in the united states, 2000–2015. Obstet. Gynecol..

[12] Moameri H., Ostadghaderi M., Khatooni E., Doosti-Irani A. (2019). Association of postpartum depression and cesarean section: A systematic review and meta-analysis. Clin. Epidemiol. Glob. Health.

[13] Özcan N.K., Boyacıoğlu N.E., Dinç H. (2017). Postpartum depression prevalence and risk factors in turkey: A systematic review and meta-analysis. Arch. Psychiatr. Nurs..

[14] Zinga D., Phillips S.D., Born L. (2005). Postpartum depression: We know the risks, can it be prevented?. Braz. J. Psychiatry.

[15] Turkcapar A.F., Kadıoğlu N., Aslan E., Tunc S., Zayıfoğlu M., Mollamahmutoğlu L. (2015). Sociodemographic and clinical features of postpartum depression among turkish women: A prospective study. BMC Pregnancy Childbirth.

[16] Hsieh C.Y., Su C.C., Shao S.C., Sung S.F., Lin S.J., Kao Yang Y.H., Lai E.C. (2019). Taiwan’s national health insurance research database: Past and future. Clin. Epidemiol..

[17] Jung C.R., Chen W.T., Tang Y.H., Hwang B.F. (2019). Fine particulate matter exposure during pregnancy and infancy and incident asthma. J. Allergy Clin. Immunol..

[18] Egede L.E., Walker R.J., Bishu K., Dismuke C.E. (2016). Trends in costs of depression in adults with diabetes in the united states: Medical expenditure panel survey, 2004–2011. J. Gen. Intern. Med..

[19] Wei Y.T., Lee P.Y., Lin C.Y., Chen H.J., Lin C.C., Wu J.S., Chang Y.F., Wu C.L., Guo H.R. (2020). Non-alcoholic fatty liver disease among patients with sleep disorders: A nationwide study of taiwan. BMC Gastroenterol..

[20] Sullivan G.M., Feinn R. (2012). Using effect size-or why the p value is not enough. J. Grad. Med. Educ..

[21] Smorti M., Ponti L., Pancetti F. (2019). A comprehensive analysis of post-partum depression risk factors: The role of socio-demographic, individual, relational, and delivery characteristics. Front. Public Health.

[22] Goker A., Yanikkerem E., Demet M.M., Dikayak S., Yildirim Y., Koyuncu F.M. (2012). Postpartum depression: Is mode of delivery a risk factor?. ISRN Obstet. Gynecol..

[23] O’Hara M.W., McCabe J.E. (2013). Postpartum depression: Current status and future directions. Annu. Rev. Clin. Psychol..

[24] Yu Y., Liang H.F., Chen J., Li Z.B., Han Y.S., Chen J.X., Li J.C. (2021). Postpartum depression: Current status and possible identification using biomarkers. Front. Psychiatry.

[25] Creedy D.K., Shochet I.M., Horsfall J. (2000). Childbirth and the development of acute trauma symptoms: Incidence and contributing factors. Birth.

[26] Ram D., Gandotra S. (2015). Antidepressants, anxiolytics, and hypnotics in pregnancy and lactation. Indian J. Psychiatry.

[27] Pearlstein T. (2013). Use of psychotropic medication during pregnancy and the postpartum period. Womens Health.

[28] Raffi E.R., Nonacs R., Cohen L.S. (2019). Safety of psychotropic medications during pregnancy. Clin. Perinatol..

[29] Suzumori N., Ebara T., Tamada H., Matsuki T., Sato H., Kato S., Saitoh S., Kamijima M., Sugiura-Ogasawara M. (2021). Relationship between delivery with anesthesia and postpartum depression: The Japan environment and children’s study (jecs). BMC Pregnancy Childbirth.

[30] Edipoglu I.S., Aslan D.D. (2021). Association of postpartum depression and epidural analgesia in women during labor: An observational study. Braz. J. Anesthesiol..

[31] Lim G., Farrell L.M., Facco F.L., Gold M.S., Wasan A.D. (2018). Labor analgesia as a predictor for reduced postpartum depression scores: A retrospective observational study. Anesth. Analg..

[32] Betran A.P., Ye J., Moller A.B., Souza J.P., Zhang J. (2021). Trends and projections of caesarean section rates: Global and regional estimates. BMJ Glob. Health.

[33] Norhayati M.N., Nik Hazlina N.H., Aniza A.A., Asrenee A.R. (2016). Severe maternal morbidity and postpartum depressive symptomatology: A prospective double cohort comparison study. Res. Nurs. Health.

[34] Furuta M., Sandall J., Cooper D., Bick D. (2014). The relationship between severe maternal morbidity and psychological health symptoms at 6-8 weeks postpartum: A prospective cohort study in one english maternity unit. BMC Pregnancy Childbirth.

[35] Abe H., Sumitani M., Uchida K., Ikeda T., Matsui H., Fushimi K., Yasunaga H., Yamada Y. (2018). Association between mode of anaesthesia and severe maternal morbidity during admission for scheduled caesarean delivery: A nationwide population-based study in Japan, 2010–2013. Br. J. Anaesth..

[36] Guglielminotti J., Landau R., Daw J., Friedman A.M., Chihuri S., Li G. (2022). Use of labor neuraxial analgesia for vaginal delivery and severe maternal morbidity. JAMA Netw. Open.

[37] Soet J.E., Brack G.A., DiIorio C. (2003). Prevalence and predictors of women’s experience of psychological trauma during childbirth. Birth.

