# Supplementary material for: Association of General Anesthesia and Neuraxial Anesthesia in Caesarean Section with Maternal Postpartum Depression: A Retrospective Nationwide Population-Based Cohort Study
Source: J Pers Med. 2022 Jun 14;12(6):970. doi: 10.3390/jpm12060970 (PMC9224722; doi:10.3390/jpm12060970)
Supplement: Supplementary file 1 [file jpm-12-00970-s001.zip › jpm-1762558-supplementary.pdf]

Supplementary Table S1 Subgroup analysis for the risk of postpartum depression in propensity score matched population

Mother aged

Mother aged <25

	NSD	CS-GA	CS-NA
Depression or sleep disorders or drugs	Reference	2.06 (1.71-2.48)	1.24 (1.03-1.50)
Depression	Reference	1.21 (0.86-1.71)	0.83 (0.57-1.20)
Sleep disorder	Reference	1.30 (0.93-1.81)	1.00 (0.70-1.42)
Drug for hypnotic and antidepressant	Reference	2.20 (1.82-2.67)	1.29 (1.06-1.57)

Mother aged 25-29

	NSD	CS-GA	CS-NA
Depression or sleep disorders or drugs	Reference	2.23 (2.00-2.49)	1.68 (1.51-1.88)
Depression	Reference	1.32 (1.06-1.65)	1.28 (1.02-1.59)
Sleep disorder	Reference	1.04 (0.87-1.24)	0.98 (0.82-1.17)
Drug for hypnotic and antidepressant	Reference	2.42 (2.16-2.72)	1.83 (1.63-2.06)

Mother aged 30-34

	NSD	CS-GA	CS-NA
Depression or sleep disorders or drugs	Reference	1.98 (1.84-2.13)	1.53 (1.42-1.66)
Depression	Reference	1.09 (0.94-1.27)	0.98 (0.84-1.15)
Sleep disorder	Reference	0.96 (0.85-1.08)	0.99 (0.87-1.12)
Drug for hypnotic and antidepressant	Reference	2.18 (2.02-2.36)	1.66 (1.53-1.80)

Mother aged 35-39

	NSD	CS-GA	CS-NA
Depression or sleep disorders or drugs	Reference	2.36 (2.16-2.58)	1.60 (1.46-1.76)
Depression	Reference	1.05 (0.88-1.25)	1.22 (1.03-1.45)
Sleep disorder	Reference	1.06 (0.92-1.22)	1.08 (0.94-1.23)
Drug for hypnotic and antidepressant	Reference	2.73 (2.48-3.00)	1.73 (1.57-1.90)

Mother aged >=40

	NSD	CS-GA	CS-NA
Depression or sleep disorders or drugs	Reference	2.24 (1.88-2.67)	1.66 (1.39-1.98)
Depression	Reference	0.86 (0.64-1.16)	0.73 (0.53-0.99)
Sleep disorder	Reference	1.18 (0.91-1.53)	0.96 (0.73-1.27)
Drug for hypnotic and antidepressant	Reference	2.37 (1.98-2.85)	1.73 (1.44-2.09)

Father aged

Father aged <25

	NSD	CS-GA	CS-NA
Depression or sleep disorders or drugs	Reference	2.00 (1.46-2.74)	1.30 (0.94-1.78)
Depression	Reference	0.67 (0.35-1.26)	0.77 (0.42-1.43)
Sleep disorder	Reference	1.34 (0.73-2.48)	1.15 (0.62-2.16)
Drug for hypnotic and antidepressant	Reference	2.04 (1.48-2.82)	1.32 (0.95-1.84)

Father aged 25-29

	NSD	CS-GA	CS-NA
Depression or sleep disorders or drugs	Reference	2.18 (1.88-2.53)	1.48 (1.27-1.73)
Depression	Reference	1.33 (1.00-1.77)	1.01 (0.75-1.37)
Sleep disorder	Reference	1.10 (0.86-1.41)	0.80 (0.62-1.05)
Drug for hypnotic and antidepressant	Reference	2.34 (2.00-2.73)	1.63 (1.39-1.91)

Father aged 30-34

	NSD	CS-GA	CS-NA
Depression or sleep disorders or drugs	Reference	2.15 (1.97-2.35)	1.68 (1.53-1.84)
Depression	Reference	1.05 (0.87-1.27)	1.20 (1.00-1.44)
Sleep disorder	Reference	1.05 (0.91-1.21)	1.04 (0.90-1.20)
Drug for hypnotic and antidepressant	Reference	2.39 (2.18-2.62)	1.81 (1.64-1.99)

Father aged 35-39

	NSD	CS-GA	CS-NA
Depression or sleep disorders or drugs	Reference	2.36 (2.16-2.59)	1.66 (1.51-1.82)
Depression	Reference	1.17 (0.98-1.41)	1.13 (0.94-1.36)
Sleep disorder	Reference	1.07 (0.92-1.24)	1.15 (1.00-1.34)
Drug for hypnotic and antidepressant	Reference	2.67 (2.43-2.95)	1.75 (1.59-1.93)

Father aged >=40

	NSD	CS-GA	CS-NA
Depression or sleep disorders or drugs	Reference	2.35 (2.08-2.64)	1.65 (1.46-1.86)
Depression	Reference	0.99 (0.79-1.24)	0.99 (0.79-1.24)
Sleep disorder	Reference	1.02 (0.85-1.23)	1.00 (0.83-1.20)
Drug for hypnotic and antidepressant	Reference	2.57 (2.27-2.91)	1.72 (1.52-1.95)

Gestational age

Gestational age <28 weeks

	NSD	CS-GA	CS-NA
Depression or sleep disorders or drugs	Reference	1.95 (1.59-2.39)	1.06 (0.85-1.32)
Depression	Reference	0.88 (0.62-1.25)	0.71 (0.47-1.07)
Sleep disorder	Reference	0.88 (0.65-1.18)	0.69 (0.49-0.97)
Drug for hypnotic and antidepressant	Reference	2.37 (1.92-2.91)	1.18 (0.94-1.49)

Gestational age 28-32 weeks

	NSD	CS-GA	CS-NA
Depression or sleep disorders or drugs	Reference	2.38 (1.98-2.87)	1.56 (1.29-1.88)
Depression	Reference	1.06 (0.74-1.52)	1.04 (0.72-1.50)
Sleep disorder	Reference	1.15 (0.84-1.57)	0.98 (0.71-1.36)
Drug for hypnotic and antidepressant	Reference	2.47 (2.04-2.98)	1.62 (1.33-1.96)

Gestational age 32-37 weeks

	NSD	CS-GA	CS-NA
Depression or sleep disorders or drugs	Reference	2.15 (1.93-2.39)	1.58 (1.41-1.76)
Depression	Reference	1.01 (0.83-1.23)	1.11 (0.92-1.36)
Sleep disorder	Reference	0.99 (0.84-1.17)	1.01 (0.86-1.19)
Drug for hypnotic and antidepressant	Reference	2.36 (2.11-2.65)	1.65 (1.47-1.85)

Gestational age 37-42 weeks

	NSD	CS-GA	CS-NA
Depression or sleep disorders or drugs	Reference	2.10 (1.99-2.23)	1.60 (1.51-1.70)
Depression	Reference	1.13 (1.01-1.27)	1.05 (0.94-1.18)
Sleep disorder	Reference	1.02 (0.93-1.12)	1.03 (0.93-1.13)
Drug for hypnotic and antidepressant	Reference	2.33 (2.19-2.47)	1.73 (1.63-1.84)

Gestational age >=42 weeks

	NSD	CS-GA	CS-NA
Depression or sleep disorders or drugs	Reference	1.69 (0.35-8.22)	0.68 (0.12-3.87)
Depression	Reference	-	-
Sleep disorder	Reference	-	-
Drug for hypnotic and antidepressant	Reference	1.85 (0.34-10.05)	0.61 (0.08-4.41)

Infant’s birth weight

Infant’s birth weight <1000 g

	NSD	CS-GA	CS-NA
Depression or sleep disorders or drugs	Reference	1.88 (1.53-2.31)	1.09 (0.89-1.35)
Depression	Reference	0.76 (0.53-1.09)	0.73 (0.50-1.06)
Sleep disorder	Reference	0.92 (0.69-1.22)	0.65 (0.47-0.90)
Drug for hypnotic and antidepressant	Reference	2.23 (1.81-2.75)	1.26 (1.01-1.56)

Birth weight 1000-1500 g

	NSD	CS-GA	CS-NA
Depression or sleep disorders or drugs	Reference	2.02 (1.66-2.46)	1.38 (1.13-1.68)
Depression	Reference	0.91 (0.62-1.34)	1.02 (0.71-1.49)
Sleep disorder	Reference	0.87 (0.62-1.22)	0.89 (0.63-1.24)
Drug for hypnotic and antidepressant	Reference	2.21 (1.81-2.71)	1.48 (1.21-1.81)

Birth weight 1500-2500 g

	NSD	CS-GA	CS-NA
Depression or sleep disorders or drugs	Reference	2.22 (2.00-2.46)	1.61 (1.45-1.79)
Depression	Reference	1.14 (0.95-1.38)	1.00 (0.82-1.20)
Sleep disorder	Reference	0.98 (0.83-1.15)	1.00 (0.86-1.17)
Drug for hypnotic and antidepressant	Reference	2.44 (2.19-2.71)	1.67 (1.50-1.86)

Birth weight 2500-3500 g

	NSD	CS-GA	CS-NA
Depression or sleep disorders or drugs	Reference	2.17 (2.04-2.31)	1.65 (1.55-1.76)
Depression	Reference	1.17 (1.03-1.32)	1.15 (1.02-1.31)
Sleep disorder	Reference	1.10 (0.99-1.22)	1.08 (0.98-1.20)
Drug for hypnotic and antidepressant	Reference	2.42 (2.26-2.59)	1.80 (1.68-1.93)

Birth weight >=3500 g

	NSD	CS-GA	CS-NA
Depression or sleep disorders or drugs	Reference	2.14 (1.87-2.45)	1.44 (1.25-1.66)
Depression	Reference	0.99 (0.74-1.32)	0.92 (0.69-1.24)
Sleep disorder	Reference	0.95 (0.76-1.19)	0.94 (0.75-1.19)
Drug for hypnotic and antidepressant	Reference	2.32 (2.01-2.69)	1.51 (1.30-1.75)

Urban

Urban

	NSD	CS-GA	CS-NA
Depression or sleep disorders or drugs	Reference	2.20 (2.07-2.33)	1.59 (1.50-1.69)
Depression	Reference	1.15 (1.02-1.30)	1.12 (0.99-1.26)
Sleep disorder	Reference	1.08 (0.98-1.19)	1.04 (0.94-1.14)
Drug for hypnotic and antidepressant	Reference	2.43 (2.28-2.59)	1.71 (1.60-1.82)

Sub-urban

	NSD	CS-GA	CS-NA
Depression or sleep disorders or drugs	Reference	2.20 (2.02-2.39)	1.62 (1.49-1.76)
Depression	Reference	0.98 (0.84-1.16)	0.93 (0.79-1.10)
Sleep disorder	Reference	0.98 (0.86-1.12)	0.99 (0.86-1.13)
Drug for hypnotic and antidepressant	Reference	2.46 (2.25-2.69)	1.78 (1.63-1.95)

Rural

	NSD	CS-GA	CS-NA
Depression or sleep disorders or drugs	Reference	1.68 (1.42-1.98)	1.21 (1.02-1.44)
Depression	Reference	1.17 (0.86-1.59)	1.10 (0.81-1.50)
Sleep disorder	Reference	0.89 (0.68-1.18)	0.90 (0.68-1.19)
Drug for hypnotic and antidepressant	Reference	1.84 (1.55-2.18)	1.25 (1.05-1.49)

Insurance amount

Insurance amount <20000

	NSD	CS-GA	CS-NA
Depression or sleep disorders or drugs	Reference	2.12 (1.97-2.29)	1.55 (1.44-1.68)
Depression	Reference	1.14 (1.00-1.31)	0.96 (0.83-1.10)
Sleep disorder	Reference	1.08 (0.96-1.22)	1.01 (0.89-1.14)
Drug for hypnotic and antidepressant	Reference	2.27 (2.10-2.46)	1.64 (1.51-1.78)

Insurance amount 20000-50000

	NSD	CS-GA	CS-NA
Depression or sleep disorders or drugs	Reference	2.18 (2.05-2.32)	1.57 (1.48-1.68)
Depression	Reference	1.09 (0.95-1.24)	1.15 (1.01-1.31)
Sleep disorder	Reference	1.02 (0.92-1.13)	1.01 (0.91-1.12)
Drug for hypnotic and antidepressant	Reference	2.46 (2.30-2.63)	1.71 (1.60-1.83)

Insurance amount >=50000

	NSD	CS-GA	CS-NA
Depression or sleep disorders or drugs	Reference	2.05 (1.68-2.50)	1.63 (1.34-2.00)
Depression	Reference	0.84 (0.54-1.29)	1.07 (0.71-1.61)
Sleep disorder	Reference	0.87 (0.63-1.19)	1.01 (0.75-1.37)
Drug for hypnotic and antidepressant	Reference	2.51 (2.03-3.10)	1.81 (1.45-2.25)

NSD: Normal Spontaneous Delivery
CS-GA: Cesarean Section- General Anesthesia
CS-NA: Neuraxial - General Anesthesia
